# Dendritic spine density is increased on nucleus accumbens D2 neurons after chronic social defeat

**DOI:** 10.1038/s41598-020-69339-7

**Published:** 2020-07-24

**Authors:** Megan E. Fox, Antonio Figueiredo, Miriam S. Menken, Mary Kay Lobo

**Affiliations:** Department of Anatomy and Neurobiology, University of Maryland School of Medicine, 20 Penn St, HSFII Building, Rm 265, Baltimore, MD 21201 USA

**Keywords:** Emotion, Striatum, Social behaviour, Motivation, Neuroscience, Spine plasticity, Spine structure

## Abstract

Stress alters the structure and function of brain reward circuitry and is an important risk factor for developing depression. In the nucleus accumbens (NAc), structural and physiological plasticity of medium spiny neurons (MSNs) have been linked to increased stress-related and depression-like behaviors. NAc MSNs have opposing roles in driving stress-related behaviors that is dependent on their dopamine receptor expression. After chronic social defeat stress, NAc MSNs exhibit increased dendritic spine density. However, it remains unclear if the dendritic spine plasticity is MSN subtype specific. Here we use viral labeling to characterize dendritic spine morphology specifically in dopamine D2 receptor expressing MSNs (D2-MSNs). After chronic social defeat, D2-MSNs exhibit increased spine density that is correlated with enhanced social avoidance behavior. Together, our data indicate dendritic spine plasticity is MSN subtype specific, improving our understanding of structural plasticity after chronic stress.

## Introduction

Stress-related mood disorders such as Major Depression and Post Traumatic Stress affect millions of individuals world-wide. Many patients do not adequately respond to first-line treatments such as monoamine reuptake inhibitors^1^, thus there is continued effort to develop novel therapeutics with more rapid onset or reduced side effect profiles. One major obstacle in developing new compounds for treating stress-related disorders is an incomplete understanding of the basic neurobiology of chronic stress^2^.

Since psychosocial stress is the primary antecedent of illnesses such as depression^2^, a significant number of stress researchers use a mouse model of psychosocial stress termed chronic social defeat stress (CSDS). This procedure uses a “resident-intruder” approach, where the experimental intruder mouse is subjected to ten, once-daily bouts of agonistic social confrontation with an aggressive resident mouse followed by continuous sensory interaction with the resident^3^. The majority of mice (~ 70%), termed “stress-susceptible” develop social avoidance and anhedonia^4^, mimicking behavioral symptoms of stress-related illnesses in humans. Further, some of the abnormal gene expression patterns in postmortem human brains are recapitulated in stress-susceptible mice^1^. A vast preclinical and human functional imaging literature implicates disrupted brain reward circuitry in the symptomology of depression^5,6^. These changes are a consequence of cellular, molecular, and structural adaptations that lead to altered neuronal activity. Nuclei within brain reward circuitry contain many heterogeneous cell-types that undergo unique adaptations after stress. Recent work indicates molecular, structural, and physiological changes in specific cell-types drive stress-related behaviors^7–16^, but our understanding remains incomplete.

The Nucleus Accumbens (NAc) has received considerable attention in depression, concordant with its role in driving motivated behavior and integrating limbic and cortical signaling. The NAc receives dense dopaminergic input from the Ventral Tegmental Area (VTA), that signals through dopamine receptors expressed on the medium spiny projection neurons in the NAc (MSNs). MSNs comprise > 95% of cells within the NAc and are divided into two subtypes based on expression of dopamine D1 or D2 receptors (D1-MSNs and D2-MSNs, respectively)^17^. The two MSN subtypes have distinct projection targets and molecular profiles. While both NAc D1- and D2-MSNs send projections to the ventral pallidum, D1-MSNs also send projections to the VTA and substantia nigra^18^. D2-MSNs are enriched in Gpr6, enkephalin, and adenosine A2A receptors, while D1-MSNs are enriched in M4 muscarinic receptors, substance P, and dynorphin^19^. Disrupting the balance between MSN subtypes by increasing D2-MSN signaling, or decreasing D1-MSN signaling can lead to increased social avoidance and anhedonia^20^.

The NAc also integrates excitatory input from several stress-sensitive afferents including thalamus, hippocampus, and medial prefrontal cortex^21–23^. After CSDS, excitatory input is strengthened onto NAc MSNs due in part to the creation of new dendritic spines^24^. However, changes in MSN physiology after CSDS are subtype specific. Excitatory input is only increased onto D2-MSNs, and is instead weakened onto D1-MSNs^20^. Given these opposing physiological changes in MSN subtypes, and an apparent lack of spine changes on D1-MSNs^9^, we hypothesized D2-MSNs have increased dendritic spine density after CSDS. To test this, we used viral labeling of D2-MSNs with the D2-MSN specific A2A-Cre line to characterize dendritic spines in mice subjected to CSDS and find density of specific D2-MSNs dendritic spine types correlates with social avoidance behavior.

## Results

### Spine density is increased in D2-MSNs after chronic social defeat stress

**Figure 1:**
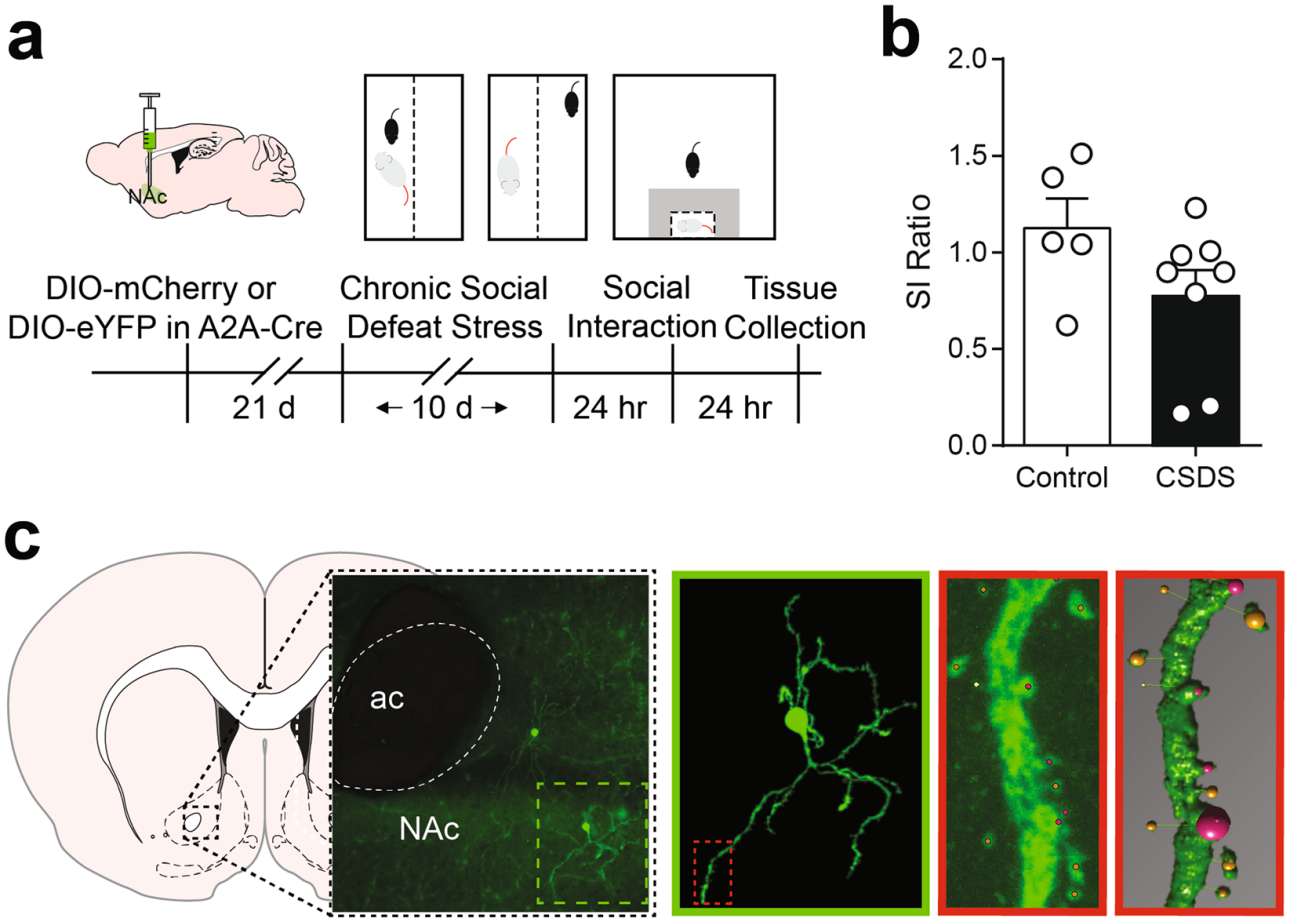
(**a**) Experimental timeline. A2A-Cre mice were injected with AAV-eYFP or AAV-mCherry to label D2-MSNs prior to chronic social defeat stress (CSDS). (**b**) Social interaction behavior after 10 days CSDS or in unstressed controls. Data points represent individual mice. (**c**) Representative image of sparsely labeled MSNs in the NAc and schematic for dendritic spine analysis workflow. Secondary dendrites of single D2-MSNs are imaged at 63x, then analyzed in Neuron Studio. Identified spines are confirmed in 3D reconstructed segments.

**Figure 2:**
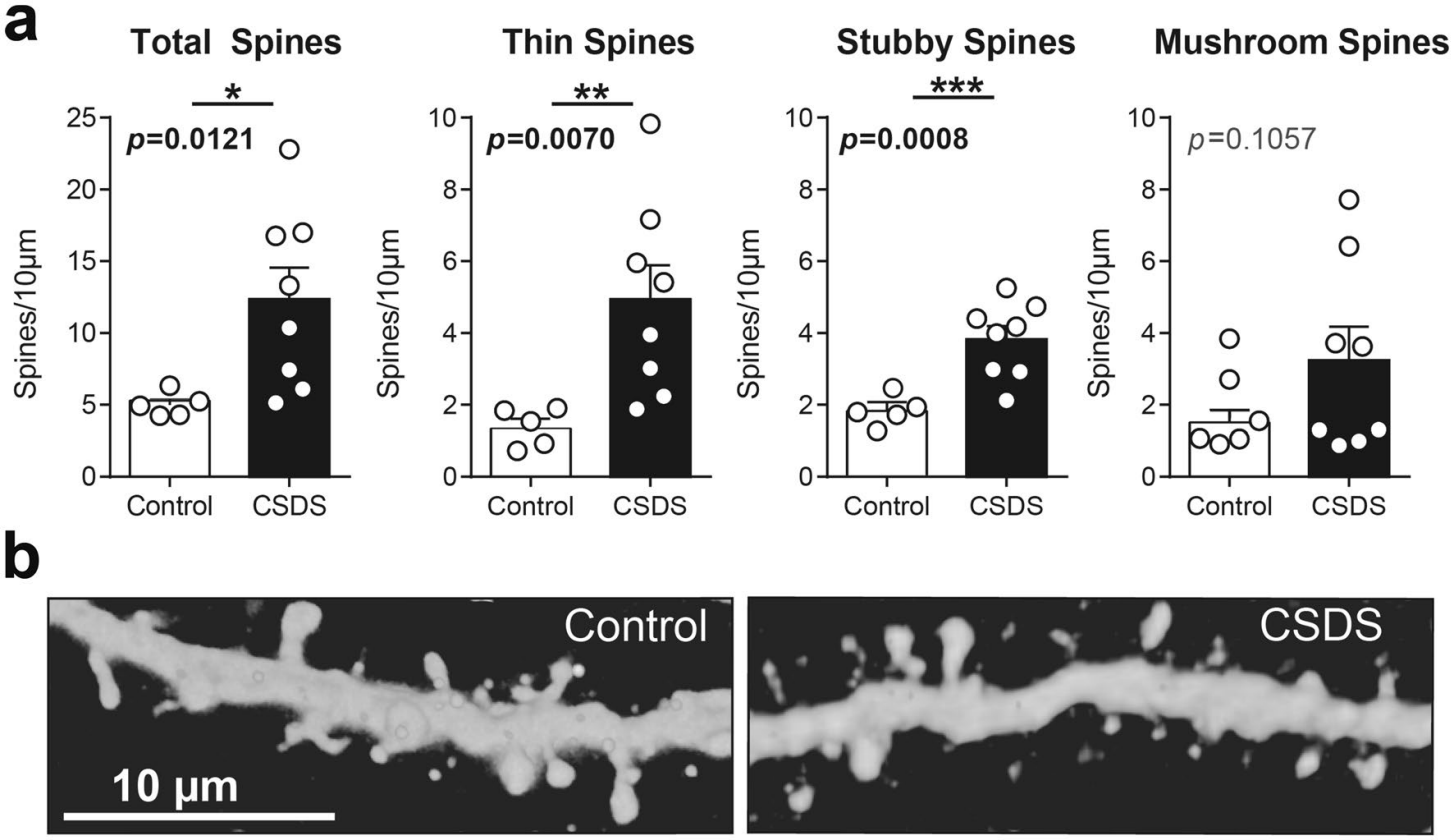
(**a**) Spine density in unstressed control and socially defeated mice, and broken down by spine type. Each individual data point represents the average of 3–4 dendrites per cell, from 3–4 cells in an individual mouse (9–15 total dendrites per mouse) (**b)** Representative dendritic segments from control and CSDS mice. *P < 0.05, **P < 0.01, ***P < 0.005, Welch’s corrected T-test.

Previous work indicates spine density is increased after CSDS^24–26^, however work from our lab indicates spine density is largely unchanged in D1-MSNs after CSDS^9^. To test the hypothesis that spine density is altered in D2-MSNs, we infused a low titer Cre dependent eYFP or mCherry to sparsely label D2-MSNs in the NAc (timeline in Fig. 1a, schematic Fig. 1c)^8^. After CSDS, mice were tested for social avoidance behavior. Due to the small number of mice, we did not divide mice into susceptible or resilient groups^4^ but instead report individual social interaction ratios. Consistent with previous work, most mice developed social avoidance after CSDS (SI ratio < 1, Fig. 1b). We assessed spine density in CSDS mice compared with unstressed controls irrespective of spine type (Fig. 2a ‘Total Spines’, representative segments in Fig. 2b). The number of spines per 10 µm dendritic length was increased in CSDS mice relative to control mice (12.4 ± 2.2 vs 5.0 ± 0.4, Welch corrected *t*_(7.418)_ = 3.297, *p* = 0.0121).

Previous work indicates CSDS increases stubby spine density^24,25^. Thus, we next assessed density of specific spine types in CSDS vs Control mice. We found increased thin spine density in CSDS mice (Fig. 2a, ‘Thin Spines’, 4.9 ± 0.9 vs 1.4 ± 0.2, Welch corrected *t*_(7.838)_ = 3.589, *p* = 0.0073), and increased stubby spine density in CSDS mice relative to control (Fig. 2a, ‘Stubby Spines’, 3.8 ± 0.4 vs 1.8 ± 0.2, Welch corrected *t*_(9.954)_ = 4.734, *p* = 0.0008). Mushroom spine density was unchanged by CSDS, although there was a trend towards increased density in CSDS mice (Fig. 2a, ‘Mushroom Spines’, 3.2 ± 0.9 vs 1.5 ± 0.3, Welch corrected *t*_(8.637)_ = 1.806, *p* = 0.1057).

### Spine density correlates with social avoidance

**Figure 3:**
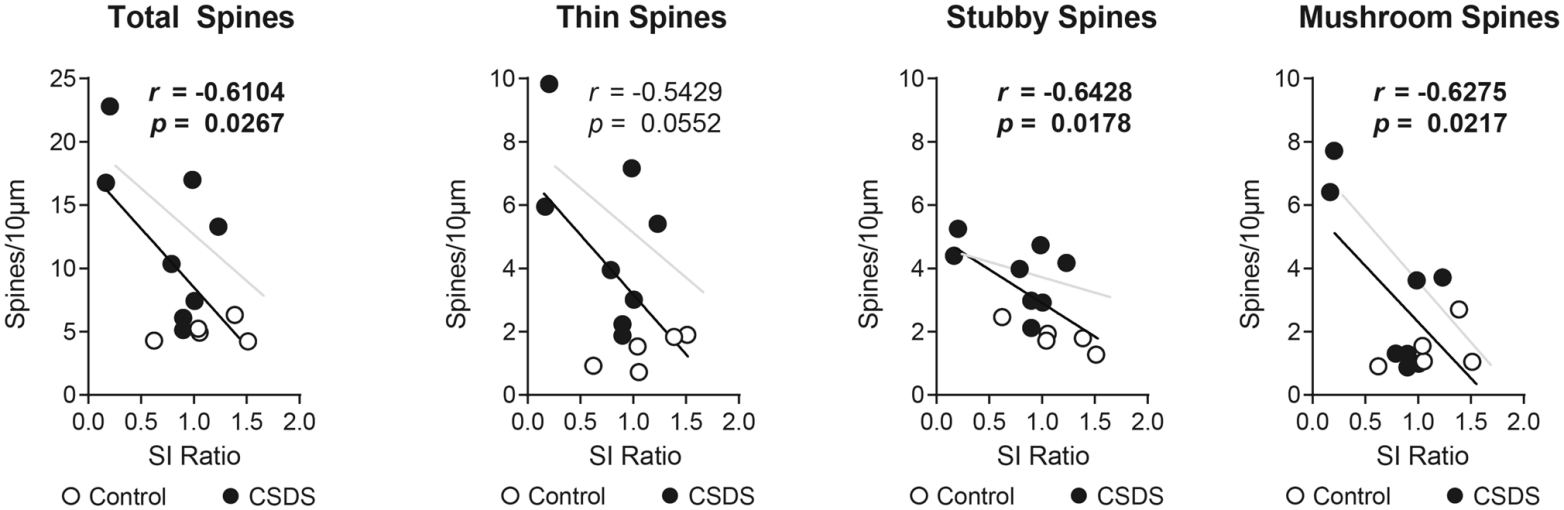
Correlations between social interaction behavior and spine density in control (white circles) and CSDS mice (black circles), and broken down by spine type. Black lines and inset R and P-values are the linear regression of both CSDS and control mice together. Gray lines are the regression of CSDS mice alone. SI Ratio is time spent in the interaction zone of a social interaction test with a novel target present divided by the time when novel target is absent.

To strengthen the argument that increased spine density after CSDS is driven by D2-MSNs, we next sought to replicate correlations between social avoidance and spine density. Using Pearson’s Correlation, we found a negative correlation between total spine density and social interaction ratio (Fig. 3, ‘Total Spines’, *r* = − 0.6104, *p* = 0.0267). When we performed similar correlations based on spine type, all three spine types showed negative correlations, with stubby and mushroom spines reaching statistical significance (Black lines in Fig. 3, ‘Thin Spines’, *r* = − 0.5429, *p* = 0.0552; ‘Stubby Spines’, *r* = − 0.6428, *p* = 0.0178; ‘Mushroom Spines’, *r* = − 0.6275, *p* = 0.0217). The negative correlations were still apparent when considering CSDS mice alone, although the lower statistical power resulted in mostly non-significant p values (Gray lines in Fig. 3, Total spines: *r* = − 0.6027, *p* = 0.1138 , Thin spines, *r* = − 0.5346*, p* = 0.1722; Stubby spines, *r* = − 0.4714, *p* = 0.2383; Mushroom spines, *r* = − 0.7431, *p* = 0.0346). Together this indicates increased spine density on D2-MSNs, especially stubby and mushroom spines, is associated with decreased social interaction.

## Discussion

This work establishes that dendritic spine density occurring after chronic social defeat stress is driven by changes to D2-MSNs. Thin and stubby spines are specifically increased on D2-MSNs of socially defeated mice, and increased spine density negatively correlates with social interaction behavior.

Stress-induced structural plasticity occurs in neurons throughout the brain, with some regions displaying consistent dendritic atrophy and dendritic spine loss following stress (e.g. hippocampus), while others exhibit increased dendritic arborization or spine density dependent on subregion (e.g. amygdala)^24–39^. In the NAc, structural plasticity is cell subtype specific, with D1-MSNs undergoing loss of dendritic arborization, but no change in spine density^8,9^. Because spine density was unchanged in D1-MSNs, we sought to confirm previously reported dendritic spine changes were present in D2-MSNs. Our findings are concordant with non-subtype specific work showing increased spine density in mice displaying increased social avoidance^24,25,40^.

In addition to overall spine density, individual dendritic spine morphology is important to consider since spine size and type are important determinants of synaptic strength^41,42^. While dendritic spine shape can change on a second-by-second basis^43^, changes to spine volume or type occur over days to months, and require several structural refinements^44^. Spines are classified into subtype based on the relative appearance of spine head and neck. “Thin” spines have long, thin necks and small heads, “mushroom” spines have thicker necks ending in large, bulbous heads, while “stubby” spines consist mainly of a spine head. Thin and stubby are considered immature spine types and are less stable as compared with the more mature mushroom type. Compared with thin and stubby, mushroom spines also form larger post synaptic densities that may translate to stronger synapses^45^. Here, we found significantly increased stubby spine density after CSDS that correlated with decreased social interaction behavior, consistent with non-subtype specific work^24,25^. Despite the smaller post synaptic density, stubby spines lack a defined neck and are more tightly coupled to their parent dendrite^46^. Thus, a change in stubby spine number may exert a proportionally greater influence on neuronal physiology. Excitatory input onto these spines may drive more action potentials in D2-MSNs, in turn driving stress-susceptibility^20^. We also found thin spine density was increased after CSDS that trended towards correlation with social avoidance (p = 0.055). The increased immature spine density after CSDS, together with the overall increase in spine density, indicates new spines are being formed on D2-MSNs—i.e. the increase in thin and stubby spine types is not due to the conversion of mushroom spines to another spine type. Indeed, mushroom spine density is trending towards an increase after CSDS, not a decrease, and we found a significant correlation between mushroom spine density and social avoidance. Thus, the most CSDS-susceptible mice may have more thin and stubby spines prior to stress that mature into mushroom spines during stress, in addition to newly created spines. As a corollary, CSDS-resilient mice may have lower spine density at baseline, leaving them less vulnerable to the negative consequence of new D2-MSN spines. Alternatively, resilient mice may upregulate synaptic pruning machinery to counteract the formation of immature dendritic structures, and/or decrease the synaptic strength of individual spines^47^. Regardless, decreasing overall D2-MSN spine strength and density is likely a key mechanism of CSDS-resilience. Future work tracking individual dendritic spine formation and stability in vivo will be critical for understanding specific mechanisms of chronic stress.

Aside from altered synaptic inputs, neuronal morphology changes are also driven by altered gene expression. For example, expression of cytoskeleton remodeling molecules is altered by CSDS^8,9,25^. While RhoA driven decreased D1-MSN dendritic complexity is sufficient to drive CSDS susceptibility^8,48^, the specific molecular mechanisms underlying increased spine formation on D2-MSNs remains unknown. It is tempting to speculate molecules with established, non-subtype specific roles, exert their effects preferentially in D2-MSNs. For example, both Dnmt3a overexpression^49^ or constitutively active IκB kinase (IKK)^40^ increase MSN spine density. Further work is needed to identify the specific molecular mechanisms for driving changes to dendritic spines specifically in D2-MSNs, and if spine changes alone are sufficient to confer susceptibility to CSDS.

In conclusion, we identified the specific MSN subtype that undergoes dendritic spine plasticity after CSDS. Since D1- and D2-MSNs undergo such opposing physiological and structural adaptations, this finding has important implications for interpreting non cell-type specific studies. Future work should continue to evaluate cellular and molecular changes after CSDS in the NAc with cell subtype in mind, which will ultimately increase our understanding of neurobiological processes underlying disrupted behaviors after stress, which has relevance for affective disorders such as depression.

## Methods

### Experimental subjects

All experiments were approved by the Institutional Animal Care and Use Committee at the University of Maryland School of Medicine (UMSOM), and performed in accordance with NIH guidelines for the use of laboratory animals. All mice were given food and water ad libitum and housed in UMSOM animal facilities on a 12:12 h light:dark cycle. Male hemizygous A2A-Cre mice (RRID:MMRRC_031168-UCD, 8–10 weeks old) were used for dendritic spine analysis after CSDS. Male CD-1 retired breeders (Charles River, > 4 months RRID:IMSR_CRL:022) were used as the aggressors for CSDS. Mice were randomly assigned to the control or stressed group.

### Stereotaxic surgery

Mice were anesthetized with isoflurane (4% induction, 1.5% maintenance) and affixed in a stereotaxic frame (Kopf Instruments). An incision was made in the scalp, and holes were drilled to target the NAc (AP + 1.6 mm, ML: ± 1.5 mm, DV: − 4.4 mm,10°). 300 nL of a Cre-inducible, double inverted open (DIO) reading frame adeno-associated virus (AAV) was infused bilaterally with Neuro Syringes (Hamilton), and the scalp closed with Vet Bond (3 M). One half of mice received AAV5-Ef1a-DIO-eYFP, and the other half received AAV2-EF1a-DIO-mCherry (UNC Vector Core, Chapel Hill, NC, USA, both diluted to 1.5 × 10^11^ VP/mL)^8^. An equal number of mice per virus was present in each group (3 control, 4 CSDS) and we combined them for dendritic spine analysis.

### Immunostaining

Mice were transcardially perfused with 0.1 M PBS and 4% paraformaldehyde. Brains were removed, post-fixed for 24 h, and 100 µm sections were collected in PBS with a vibratome (Leica, Germany). Immunostaining was performed according to our previously published procedures^8^. Briefly, slices were washed with PBS and blocked in 3% normal donkey serum (NDS) with 0.3% Triton X-100. Slices were incubated in chicken anti-GFP (1:500; Aves Lab, Tigard, OR, USA; RRID:AB_10000240) or chicken anti-mCherry (1:1,000; Novus Biologicals, Centennial, CO, USA; RRID:AB_2636881) at 4 °C overnight in the blocking buffer. Slices were washed in PBS, then incubated in Anti-Chicken Alexa 488 (1:500; Jackson Immuno, West Grove, PA, USA; RRID:AB_2338052) or Anti-Chicken Cy3 (1:500, Jackson Immuno; RRID:AB_2340363) at 4 °C overnight. Slices were washed with PBS, then mounted with Vectashield mounting media (RRID:AB_2336789), and imaged on a laser-scanning confocal microscope (Leica SP8, RRID:SCR_018169).

### Dendritic spine analysis

Sections containing NAc were sampled from bregma AP: 1.5–1.0 mm and Z-stack images were acquired at 0.2 µm increments using a 63 × objective (1.4 NA, pixel size 86.1 × 86.1 nm). We imaged secondary dendrites from 3–4 cells per mouse from at least 2 separate slices in both NAc core and shell, including cells on the core/shell border (See Fig. 1a). Using Neuron Studio software^50^ (Mt Sinai School of Medicine), a blinded experimenter selected secondary dendrites (> 20 µm long, > 40 µm from soma) that could be clearly resolved from neighboring dendrites (i.e. no overlapping branches)^24,25,51–53^. This resulted in a different number of dendrites available for analysis in each cell, thus 3–4 of these dendrites per cell were randomly selected, yielding a total of 9–15 dendrites per mouse. We analyzed secondary dendrites due to the relative dearth of spines on the more proximal, primary dendrites of MSNs, and to be consistent with existing literature^24,25,40,52,54^. To reduce background noise, the Z-blur and median blur filters were applied to all images. All spines identified by Neuron Studio were visually confirmed in a 3D rendering by a blinded experimenter, and any misidentified spines (e.g. noise, dendritic segment) were manually removed. Spine densities were first averaged for a given cell, and then all cells from the mouse were averaged together to generate a ‘grand average’ for an individual mouse. If a sufficient number of dendrites could not be analyzed from a given mouse it was removed from the study. One control mouse was removed from the main figures with Grubb’s Outlier Test (α = 0.05). Figures with the outlier included are available in Supplemental Fig. 1.

### Social defeat stress

Chronic social defeat stress (CSDS) was performed according to well-documented procedures^3^ as previously in the lab^8–10,48,55^. The mice were placed in hamster cages with perforated plexiglass dividers containing a novel, aggressive CD1-resident. Mice were physically defeated by a new resident for 10 min, then housed opposite the resident for 24 h sensory interaction for 10 consecutive days, each day encountering a new aggressive resident. The unstressed control mice were housed 2/cage, separated by a perforated plexiglass divider. 24 h after the last defeat, social avoidance was assessed with videotracking software (CleverSys, Reston, VA, USA). Experimental mice were placed in an open field containing a perforated chamber. Time spent around the chamber (“interaction zone”) was compared between two, 2.5 min trials during which the chamber was empty or contained a novel CD-1. Social interaction ratios were calculated by dividing time spent in the interaction zone with and without the novel mouse present.

### Statistics

All statistics were performed using Graph Pad Prism software. Only mice for which we successfully analyzed 9–15 dendrites are included in this report. Unpaired two-tailed t-tests with Welch’s correction for unequal variance were used to compare between control and CSDS spine densities. Pearson’s correlation was used to compare the relationship between spine density and social interaction ratio.

## Supplementary information

Supplementary Information.

## Data Availability

The datasets generated during and/or analyzed during the current study are available from the corresponding author on reasonable request.
