# Supplementary material for: Dendritic spine density is increased on nucleus accumbens D2 neurons after chronic social defeat
Source: Sci Rep. 2020 Jul 24;10:12393. doi: 10.1038/s41598-020-69339-7 (PMC7381630; doi:10.1038/s41598-020-69339-7)
Supplement: Supplementary file 1 — Supplementary Information. [file 41598_2020_69339_MOESM1_ESM.docx]

**Dendritic spine density is increased on nucleus accumbens D2 neurons after chronic social defeat**

Megan E. Fox^1^, Antonio Figueiredo^1#^, Miriam S. Menken^1#^, Mary Kay Lobo^1^*

1. Department of Anatomy and Neurobiology, University of Maryland School of Medicine, Baltimore, MD, USA

# These authors contributed equally

*Corresponding Author

Mary Kay Lobo

20 Penn St

HSFII Building, Rm 265

Baltimore, MD 21201

Tel: 410-706-8824

Email: [mklobo@som.umaryland.edu](mailto:mklobo@som.umaryland.edu)


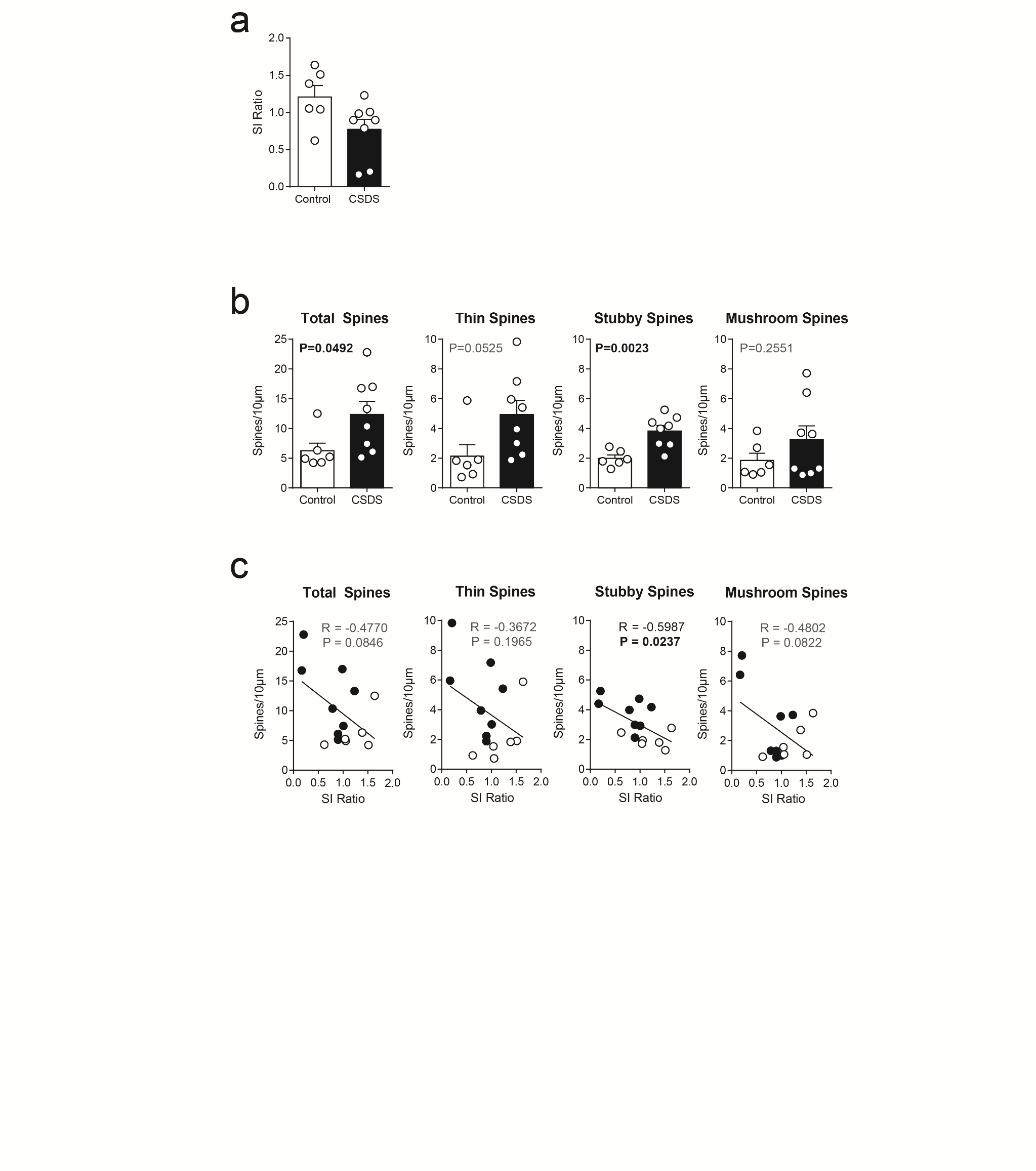


**Supplemental Figure 1.** Data including the outlier control mouse removed with Grubb’s test. (**a**) Compare with Fig 1 b. Social Interaction (SI) ratio in control and CSDS mice. (**b**) Compare with Fig 2a. Spine density in control and CSDS mice, broken down by individual spine type. Each data point represents the average of 9-15 dendrites from an individual mouse (3-4 dendrites from 3-4 cells per mouse). Total Spines: t_(12)_=2.188, P=0.0492, Thin: t_(12)_=3.589, P=0.0525; Stubby: t_(12)_=3.854, P=0.0023; Mushroom: t_(12)_=1.195, p=0.2551 (**c**) Compare with Fig 3. Pearson’s correlation between spine density in control (white) and CSDS (black) mice, broken down by individual spine type.
